# The Evolutionary Innovation of Nutritional Symbioses in Leaf-Cutter Ants

**DOI:** 10.3390/insects3010041

**Published:** 2012-01-06

**Authors:** Frank O. Aylward, Cameron R. Currie, Garret Suen

**Affiliations:** 1 Department of Bacteriology, Microbial Sciences Building, 1550 Linden Drive, University of Wisconsin-Madison, Madison, WI 53706, USA; E-Mails: faylward@wisc.edu (F.O.A.); currie@bact.wisc.edu (C.R.C.); 2 Department of Energy Great Lakes Bioenergy Research Center, 1550 Linden Drive, University of Wisconsin-Madison, Madison, WI 53706, USA

**Keywords:** symbiosis, attine ants, *Leucoagaricus*, co-evolution, microbial consortia

## Abstract

Fungus-growing ants gain access to nutrients stored in plant biomass through their association with a mutualistic fungus they grow for food. This 50 million-year-old obligate mutualism likely facilitated some of these species becoming dominant Neotropical herbivores that can achieve immense colony sizes. Recent culture-independent investigations have shed light on the conversion of plant biomass into nutrients within ant fungus gardens, revealing that this process involves both the fungal cultivar and a symbiotic community of bacteria including *Enterobacter*, *Klebsiella*, *and Pantoea* species. Moreover, the genome sequences of the leaf-cutter ants *Atta cephalotes* and *Acromyrmex echinatior* have provided key insights into how this symbiosis has shaped the evolution of these ants at a genetic level. Here we summarize the findings of recent research on the microbial community dynamics within fungus-growing ant fungus gardens and discuss their implications for this ancient symbiosis.

## 1. Introduction

Symbioses between microbes and metazoans are widespread in nature [1,2,3]. Although these associations form for a variety of reasons, often the diverse metabolic capabilities of symbiotic microbes allow host organisms to occupy ecological niches that would otherwise be unavailable. These symbioses can involve one or a few symbionts, but many associations in nature involve complex communities of microbes. The taxonomic and physiological diversity of these communities can be massive, and research has only recently shed light on the extent to which they have shaped the evolution and ecology of metazoans [2,4,5,6]. Some of the best studied examples of associations between metazoans and complex microbial communities are in herbivores, where communities of microbes have been shown to be largely responsible for the deconstruction and conversion of recalcitrant plant material into nutrients for their hosts. Symbiotic microbial communities that provide this service have been shown to be associated with a vast array of hosts, including insects, mammals, and even molluscs [4,7,8,9,10]. 

The association between attine ants and their fungus gardens is a paradigmatic example of symbiosis between herbivores and microbial communities. Thought to have originated 45–50 million years ago in the Amazon basin [11], the symbiosis between these ants and their symbiotic fungus allowed for the subsequent diversification into >230 species ranging from Argentina to the shores of New Jersey in the USA [12,13,14]. Although many species form small colonies of only a few dozen ant workers, the most derived species, the leaf-cutter ants, have evolved to become dominant Neotropical herbivores capable of foraging on up to 17% of the foliar biomass in some ecosystems [15]. Moreover, the size of attine colonies varies dramatically, with the largest containing upwards of 8 million ants, 7 orders of magnitude more than colonies of the smallest species [14,16]. The diversity of these ants and their symbiosis with fungus garden communities have made them a model system for the study of the ecological and evolutionary implications of symbiosis. 

In this review, we focus on recent research on the association between fungus-growing ants and their fungus gardens, with an emphasis on how the evolution and ecology of the organisms in this system have been shaped through symbiosis. We pay particular attention to how the microbial communities associated with these ants mediate the deconstruction and conversion of plant biomass into usable energy. Furthermore, we discuss how the recently sequenced genomes of two leaf-cutter ant species provide insight into how this ancient symbiosis has impacted the ants on a genetic and physiological level [17,18]. Finally, we discuss how our view of this system has changed with the recent discovery of additional microbial symbionts, and suggest future avenues of research that will yield novel insights into this complex symbiotic system. 

## 2. The Fungus Garden Ecosystem

**Figure 1:**
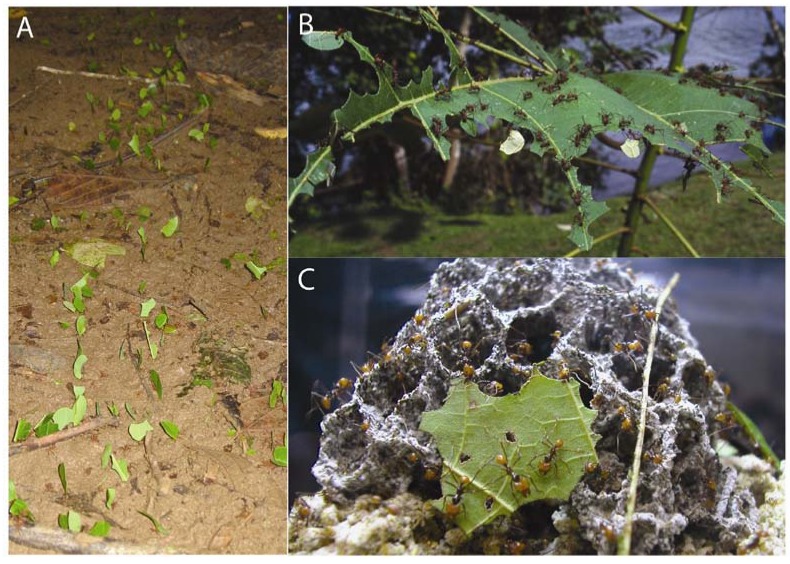
(**a, b**) Leaf-cutter ants forage on large quantities of fresh foliar biomass. (**c**) They bring this material into their subterranean nests, where it is integrated into symbiotic fungus gardens they cultivate for food. [Photo credits: A; Jarrod J. Scott, B; Christian R. Linder, used under the GNU Free Documentation License, Version 1.2, C; Austin D. Lynch.].

The most conspicuous symbiont of fungus-growing ants is the basidiomycetous fungus they grow for food. Although in the majority of species this is a lepiotaceious fungus of the genus *Leucoagaricus*, a small number of fungus-growing ant species culture a distantly-related pterulaceous group [19,20]. In most cases, the fungus is cultured by the ants on plant forage and subsequently consumed for food (Figure 1) [21,22]. The most derived group of fungus-farmers, referred to as the “higher attines”, culture a specific clade of the fungus, among which is the well-studied species *Leucoagaricus gongylophorus*. This fungus produces nutrient-rich hyphal swellings, called gongylidia, which nourish the queen and brood of a colony [19,23]. The less-derived groups, or “lower attines”, culture a broader range of fungal symbionts, and appear to have re-acquired cultivars from the environment multiple times in the course of their evolutionary history [19,24]. 

Only recently have efforts been focused on characterizing microbes in this ecosystem other than the fungal cultivar. Fungus gardens of leaf-cutter ants have so far been found to contain numerous microbial groups in addition to the dominant fungal mutualist (Table 1). The most well-described symbionts include a specialized parasite of the fungal cultivar, *Escovopsis*, as well as an antibiotic-producing Actinobacterium (genus *Pseudonocardia*) found to defend against it [25,26,27]. Research on these two symbionts has previously been reviewed [28,29], and will be discussed here only briefly. Numerous other microfungi and yeasts have also been found associated with the fungus gardens of many ant species [30,31,32,33,34,35,36,37,38]. While filamentous fungi likely represent garden “weeds” [34,39], it is unclear if the yeasts have a deleterious effect on the fungus garden ecosystem. One study even found evidence that yeasts may antagonize microfungal pests [33]. Of the bacteria cultured from fungus gardens, many have been proposed to have important roles in the fungus garden ecosystem ranging from antibiotic-mediated exclusion of pathogens to nutrient biosynthesis [40,41,42]. However, the consistent presence of many of these microbes has yet to be demonstrated, and it remains a possibility that they represent allochthonous groups introduced from the incoming foliar material or surrounding soil. 

Recently, culture-independent techniques have begun to shed light on bacterial groups thought to be common constituents of fungus gardens (Table 1). Membrane lipid profiles have shown that different leaf-cutter ant fungus gardens are highly similar, and that Gram-negative bacteria likely dominate the prokaryotic component of these ecosystems [43]. Subsequent 16S libraries have confirmed this, and further indicated that γ-proteobacteria are particularly diverse in these environments, although sequences matching to α-, β- and δ-Proteobacteria, Bacteriodetes, Firmicutes, Actinobacteria, Acidobacteria, and several other phyla were also recovered [44]. The most recent metagenomic investigation of leaf-cutter ant fungus gardens has indicated that γ-proteobacteria in the genera *Enterobacter*, *Pantoea*, *Klebsiella*, *Citrobacter*, and *Escherichia* may constitute a core prokaryotic community [45]. In addition to culture-independent work, one study successfully cultured *Klebsiella* and *Pantoea* isolates from a variety of leaf-cutter ant nests and demonstrated their capacity to fix nitrogen [46]. Moreover, this study traced nitrogen in fungus gardens to the biomass of the ants themselves, indicating that bacteria could be playing an important nutritional role in these ecosystems. Nitrogen fixation was proposed to be a critical process in fungus gardens given the relatively high nitrogen content of ant biomass compared to the incoming plant forage and the surrounding ecosystem. 

## 3. The Ancillary Gut Hypothesis

Some authors have postulated that the fungus garden ecosystem serves as an external gut for the entire ant colony [44,45,46]. Fungus-growing ants have previously been described as a “superorganism” in that the worker castes are not reproductively viable, and survival of the colony hinges completely on the queen [14]. Given that colonies of fungus growing ants are superorganisms rather than conglomerations of individuals, it follows that fungus gardens can be viewed as “organs” responsible for nutrient conversion and assimilation, similar to the digestive gut of other herbivores. 

The external gut hypothesis goes deeper than analogy. For example, both true guts and fungus gardens are specialized structures that harbor populations of bacteria that assist in the conversion of dietary material into nutrients for the host [44,47,48]. Prokaryotic populations in both true guts and fungus gardens have been implicated in the biosynthesis of nutrients for their hosts [45,46,48,49]. Perhaps the largest difference between these ecosystems is that the structural integrity of fungus gardens is provided by the fungal symbiont, whereas the prokaryotic component of true guts is harbored directly within the host. Moreover, the fungus garden ecosystem is dominated by the fungal cultivar, whereas true guts have a relatively smaller and more diverse fungal component [50,51,52]. 

Non-taxonomic similarities in prokaryotic diversity are also evident when comparing fungus gardens and mammalian guts. Low-phylum level and high strain-level diversity have been observed in both of these ecosystems when 16S ribosomal genes have been sequenced [4,5,44,45,53]. This pattern is independent of microbial taxonomy, as many mammalian guts are composed of primarily Bacteriodetes and Firmicutes [4,5,8,53,54,55,56], whereas γ-proteobacteria appear to dominate the prokaryotic component of fungus gardens. The causes of this distinct diversity profile are unclear, although in mammals it has been hypothesized that it may be the result of an adaptive radiation of a few initial prokaryotic “colonists” [5]. The relatively recent origin of these symbiotic niches compared to free living environments on the planet has also been implicated in limiting the number of microbial phyla that have evolved to live in them [5]. 

**Table 1:** Recent research on microbial diversity and plant biomass degradation in attine fungus gardens.

Ant Genera	Collection Location	Microbes Analyzed	Plant Polymers Analyzed	Methods	Principle Findings	Reference
*Atta*	Brazil	*L. gongylophorus*	Cellulose	Growth assays, enzymatic assays.	Evidence that *L. gongylophorus* can grow on cellulose in pure culture and hydrolyze this polymer efficiently.	[57]
*Atta*	Brazil	Bacteria	Gelatin, cellulose, cellobiose, casein	Directed culturing.	Isolation of plant polymer-degrading bacteria from fungus gardens.	[58]
*Acromyrmex*, *Atta*, *Myrmicocrypta*, *Trachymyrmex*, *Cyphomyrmex*	Brazil, Texas, Trinidad and Tobago	Microfungi, yeasts,	NA	Culturing, bioassays.	Isolation and characterization of microfungi and yeasts from fungus gardens, especially those of the genera* Escovopsis*, *Fusarium*, and *Trichoderma; *evidence that yeasts may antagonize potential garden pathogens; Identification of a novel *Trichosporon* species in fungus gardens.	[26,27,30,31,32,33,35,37,38,39]
*Acromyrmex*, *Atta*	Argentina	*L. gongylophorus*	Cellulose	Pure culture growth assays, estimation of lignin and cellulose content.	Indication that *L. gongylophorus* does not degrade cellulose in pure culture.	[59,60]
*Acromyrmex*, *Atta*	Brazil, Panama	Whole fungus garden, ants, larvae	Polysaccharides, heterosides, oligosaccharides	Enzymatic activity assays on workers, larvae, and fungus gardens.	Enzymatic activity profiles for the fungus garden and host ants were largely non-overlapping; xylanase, amylase, laminarinase, cellulase, and lichenanase activities identified in fungus garden samples; evidence for high variability in the enzymatic activities of fungus gardens between different nests and ant species.	[61,62]
*Acromyrmex*, *Atta*	Panama	*L. gongylophorus*	Pectin, CMC, ABTS (laccase substrate), protein	Isoelectric focusing, enzymatic assays.	Identification of fungal pectinases, CMCases, proteinases, and laccases concentrated by leaf-cutter ants in their fecal droplets.	[63]
*Atta*	Brazil	*L. gongylophorus*	Starch, pectin, xylan, cellulose, CMC	Growth and enzymatic assays.	Rapid growth of *L. gongylophorus* on xylan and starch, but poor growth on cellulose; Production of pectinases, xylanases, cellulases, and amylases by *L. gongylophorus* when grown in pure culture on different carbon sources; Identification of potential mechanisms for regulation of *L. gongylophorus* starch metabolism by the ant hosts.	[64,65,66]
*Atta*	Brazil	Whole fungus garden	All biomass	Estimation of cellulose and lignin content.	Evidence that the lignin:cellulose ratio is higher in fungus garden waste than leaf material.	[67]
*Acromyrmex*	Panama	*L. gongylophorus*	Xylan	Activity measurements of a xylanase, AZCL-based colorimetric assays.	Identification and characterization of an *L. gongylophorus* xylanase.	[68]
*Acromyrmex*	Brazil	Whole fungus garden, *L. gongylophorus *pure cultures	Numerous polysaccharides	Enzymatic activity assays on *L. gongylophorus* and whole fungus gardens, SEM.	Demonstration of broad lignocellulolytic capabilities of *L. gongylophorus* and whole fungus gardens, including activity against, laminarin, chitin, pectin, and CMC.	[69]
Cross phylogeny	Panama	Whole fungus gardens	Numerous polysaccharides	AZCL-based colorimetric assays.	Evidence for an evolutionary transition towards more efficient proteinase and amylase activity in leaf-cutter ant fungus gardens; evidence for broad lignocellulolytic capacity in lower attine fungus gardens.	[70]
*Atta*	Texas, Panama	*L. gongylophorus*	NA	Microsattelite profiling of *L. gongylophorus *from different ant nests and time points.	Confirms that a single strain of *L. gongylophorus* is cultured in leaf-cutter ant fungus gardens.	[23]
*Atta*, *Acromyrmex*	Panama, Costa Rica, Argentina	*Pantoea*, *Klebsiella*	NA	Directed culturing, stable isotope analysis, acetylene reduction analysis, phylogenetic comparisons.	Identification of nitrogen-fixing *Klebsiella* and *Pantoea *isolates in fungus gardens; Evidence that nitrogen fixed in fungus gardens is integrated into ant biomass.	[46]
*Acromyrmex*	Panama	*L. gongylophorus*	Pectin	Proteomics, RT-qPCR, enzymatic assays.	Identification of diverse fungal pectinases concentrated in the fecal droplets of the ants; evidence that *L. gongylophorus* produces enzymes specifically in the hyphal swellings fed to the ants.	[71]
*Atta*, *Acromyrmex*	Panama, Argentina	Primarily Gram-negative bacteria	NA	Lipid profiling using PLFA and FAME.	Evidence that ant fungus gardens and refuse dumps contain distinct microbial communities; evidence that the prokaryotic fraction of fungus gardens is dominated by Gram-negative bacteria.	[43]
*Atta*	Panama	γ-proteobacteria, primarily *Klebsiella* and *Pantoea*	Cellulose, hemicelluloses	Community metagenomics, 16S surveys, genome sequencing, enzymatic assays, sugar composition analysis.	Survey of bacterial diversity in fungus gardens; Identification of abundant *Klebsiella* and *Pantoea* populations; characterization of bacterial glycoside hydrolases; Evidence for significant amounts of cellulose degradation in fungus gardens.	[44]
*Atta*		Whole fungus garden	Numerous polysaccharides	AZCL-based colorimetric assays	Evidence that enzyme profiles in fungus gardens shift rapidly when integrated foliar biomass changes.	[72]
*Acromyrmex*	Panama	Whole fungus garden	Pectin, xylose	Antibody and CBM-based polysaccharide microarray profiling, AZCL-based colorimetric assays.	Evidence for the degradation of xylan and pectin, but not cellulose, in fungus gardens; Indication that plant material is only partially degraded in these ecosystems.	[73]
*Atta*	Brazil	*L. gongylophorus*	All biomass	Dye and photomicrography of plant biomass.	Evidence for substantial degradation of all non-lignified plant tissues in fungus gardens; indication that *L. gongylophorus* may degrade a large quantity of cellulose in fungus gardens	[74]
Cross phylogeny	Panama	*L. gongylophorus*	Protein	Enzymatic assays, pH and buffering analysis.	Indication that the fungal cultivars of higher attines have evolved proteinases with activity optima at pH ~5, closer to the pH of fungus gardens; characterization of different proteinase classes and buffing capacities in different fungus gardens	[75]
*Atta*	Panama	Bacteria	Cellulose, hemicelluloses	Community metagenomics, 16S surveys, metaproteomics.	Identification of abundant *Enterobacter* population in fungus gardens; Further characterization of *Klebsiella* and *Pantoea* populations; Proteomic identification of bacterial glycoside hydrolases; Identification of bacteriophage in fungus gardens	[45]

## 4. Evolution of Hygiene in the Attines

The finding that fungus gardens are composed predominantly of one fungal symbiont and 5–6 bacterial groups has led to the question of how this community composition is maintained. The partially-degraded plant material present in fungus gardens could potentially be used as a substrate for countless microorganisms, yet somehow only a small number predominate. Furthermore, the composition of the fungus garden community has been shown to be relatively consistent between fungus garden strata irrespective of the extent of biomass degradation [44,45], suggesting the existence of selective pressures to maintain a consistent community.

A number of factors likely contribute to the low diversity in fungus gardens (Table 2). The meticulous cleaning of fungus gardens by the ants is likely paramount, both for maintaining healthy cultures of the fungal symbiont and a consistent prokaryotic assemblage. Three main hygienic behaviors have been documented in fungus-growing ants. The first, termed weeding, is characterized by the specific removal of whole fragments of fungus garden material [76]. The ants weed their gardens to remove dead fungal debris as well as areas infected with pests, especially the specialized parasite *Escovopsis*. Some species of leaf-cutter ants have been shown to maintain specialized waste dumps for their agricultural waste, and the separation between these refuse heaps and fungus gardens is likely key to the maintenance of overall nest hygiene. 

**Table 2:** Factors contributing to microbial assemblage composition in attine ant fungus gardens.

Factors limiting diversity	Weeding and grooming of fungus gardens, application of glandular secretions, application of antimicrobials from *Pseudonocardia*, antibiotics produced by the fungal cultivar, fecal droplets of the ants
Factors promoting diversity	Complex, nutrient rich substrate
Potential sources of microbial groups	Maternal transmission from parent colony, phyllosphere microbes on foliar biomass, surrounding soil, the ants themselves

The second behavior, termed ‘fungus grooming’, is characterized by the licking of fungus garden material by the ants and selective filtering of foreign spores into infrabuccal pockets located in their oral cavities [76]. This behavior appears to be critical for removing spores of foreign fungi that could lead to future infection. Both weeding and grooming have been stimulated in experimental ant nests by the addition of foreign fungal spores, suggesting that the ants have acute mechanisms for assessing the composition and health of their gardens [76]. 

The third behavior involves the application of fecal droplets to the fungus garden matrix. Some species of attines have been shown to concentrate fungal chitinases and lignocellulases in these droplets, and their integration into fungus gardens has been hypothesized to contribute both to plant biomass degradation and the removal of fungal pests [63,77,78]. These drastic changes in the behavior of attines highlight how the long-term maintenance of symbiosis can have profound impacts on the life history of host organisms.

Glandular secretions of the ants themselves have also been shown to play a role in maintaining the hygiene of fungus gardens. Although known to ward off infection in all ants [79,80,81], metapleural glands may also be used by fungus-growing ants to remove unwanted microbial groups from their fungus gardens. For example, studies have found that ants consistently rub their legs against their metapleural glands while weeding and grooming so as to apply glandular secretions to the fungus garden [82,83]. Moreover, the application of metapleural gland secretions increases with weeding and grooming behavior when foreign fungal spores are experimentally introduced into a nest [82,83]. Chemical analysis of leaf-cutter ant metapleural gland secretions identified phenylacetic acid and number of short-chain fatty acids known to have antimicrobial properties [84,85]. Bioassays have confirmed that these glandular secretions have broad antifungal and antibacterial activity [86,87]. Secretions of the mandibular gland have also been implicated in potentially inhibiting the germination of alien fungi [78]. Because glandular secretions could potentially inhibit the growth of the fungal cultivar if consistently introduced to fungus gardens, the ants may rely only on selective application of these secretions to areas thought to be infected. 

The fungus itself may also produce compounds that selectively inhibit or promote the growth of other microbes in its environment. Basidiomycetes in general are a rich source of secondary metabolites [88], and novel antimicrobial compounds have previously been identified from *Leucoagaricus* species [89]. Moreover, the fungus cultured by the ants has been implicated in the production of organohalogens [90], which may be involved in lignocellulose degradation or antibiosis in basidiomycetes [91]. Antibiosis of cultivated *Leucoagaricus* isolates against *Escovopsis* species has also been shown, suggesting these fungi have at least some capacity for the production of secondary metabolites [92]. 

Lastly, fungus-growing ants have been shown to constrain microbial diversity in their fungus gardens through association with antibiotic-producing Actinobacteria [25,29,42,93,94,95,96,97]. Bacteria of the genus *Pseudonocardia* have been shown to produce compounds that inhibit the specialized garden parasite *Escovopsis* [25,98], and experimental evidence suggests these microbes play a role in maintaining garden hygiene [93,94]. Other Actinobacteria isolated from ant colonies have also been proposed to play a role in the defense against garden pathogens, but the consistent presence of these microbes in attine nests has yet to be determined [42,99,100]. Regardless, the combination of compounds produced by Actinobacteria and the fungal cultivar, together with the glandular secretions of the ants, likely produces a potent antimicrobial cocktail that could be critical for shaping microbial diversity in fungus gardens.

## 5. Plant Biomass Degradation in Fungus Gardens

The conversion of plant biomass into nutrients usable by the ants is the central role of fungus gardens. Despite the central importance of this process, the mechanisms through which plant biomass is degraded in these ecosystems are only beginning to be elucidated (Table 1). In general, the plant biomass integrated into fungus gardens is a rich source of cellulose, hemicelluloses, protein, lignin, simple sugars, and various other compounds. In the fungus gardens of higher attines, this is converted into hyphal swellings called gongylidia, which are rich in lipids, carbohydrates, and other nutrients produced by the fungal cultivar [101,102]. Importantly, gongylidia serve as a primary food source for the entire ant colony, and are the exclusive nutrient source for the developing larvae and brood [14,22]. Identification of the mechanisms through which biomass is degraded, how it is converted into energy for the ants, and which microbes are taking part in these processes is critical to a fundamental understanding of the ant-fungus garden symbiosis. 

Most work has focused on the lignocellulolytic capacity of *L. gongylophorus*, the species of fungus cultured by leaf-cutter ants. Numerous growth- and enzyme-based assays have indicated that pure cultures of this organism can degrade and grow rapidly on both starch and xylan [64,65,66]. Furthermore, while pectin is degraded rapidly by this organism, this polymer supports only intermediate growth [64,66]. Numerous pectinases and one xylanase have been identified in *L. gongylophorus *[68,71], providing evidence that it possesses coding potential typical of other saprotrophic basidiomycetes. 

Analysis of whole fungus garden extract has indicated that a wide variety of plant polymers are degraded in fungus gardens, including xylan, pectin, starch, laminarin, cellulose, lichenan, and chitin [61,62,69,70,72]. One study sought to compare enzymatic profiles of fungus gardens across the phylogeny of the attines [70]. This analysis found that the fungus gardens of leaf-cutter ants had higher amylase activity than fungus gardens of the lower attines [70]. Moreover, the overall proteinase activity was significantly higher in all higher attine fungus gardens compared to the fungus gardens of lower attines [70]. Another study found that the pH optima of proteinases in leaf-cutter ant fungus gardens was lower than those in lower attine fungus gardens, and that the buffering capacity in leaf-cutter ant fungus gardens was also higher [75]. Together these results suggest that multiple evolutionary transitions throughout the history of the ant-fungus garden association have led to a specialized form of biomass degradation in leaf-cutter ant fungus gardens [70,75]. 

The most controversial aspect of plant biomass degradation in fungus gardens is the deconstruction of cellulose. Early work found support for the hypothesis that cellulose was the primary polymer supporting fungal growth in these environments, and it was estimated that up to 45% of the cellulose in foliar biomass was degraded in fungus gardens [103]. More recently, however, studies have challenged this hypothesis, reporting that *L. gongylophorus* cannot grow in pure culture with cellulose as the sole carbon source [57,59,60], suggesting that the secreted proteins of this fungus have only limited cellulolytic activity [64,66]. Measurements of cellulose degradation in fungus gardens have also varied, with sugar composition analyses indicating that ~30% of the cellulose in foliar biomass is degraded in fungus gardens [44], while a polysaccharide microarray approach documented only limited degradation of this polymer [73]. One microscopy-based study examined plant biomass in fungus gardens throughout different stages of decomposition and found strong support for the degradation of parenchyma, endodermis, and vascular bundle cells, indicating that all plant polymers in the plant cell wall, with the exception of lignin, were degraded extensively [74]. 

High variability in the lignocellulolytic activity in fungus gardens may explain some of these conflicting results. Measurements of the enzymatic activity of whole fungus garden protein extracts typically vary over a wide range, even when fungus gardens of the same species of ant are compared [62,70]. One study even documented a rapid shift in enzymatic activity in this environment when laboratory ant colonies were switched from a diet of foliar biomass to one of starch-rich rice [72]. It is thus possible that large quantities of cellulose are degraded in fungus gardens, but that this amount varies depending on the foliar biomass provided by the ants. Indeed, because such a variety of plants are harvested by leaf-cutter ants [104,105,106], it may be crucial that *L. gongylophorus* is capable of both degrading a variety of plant polymers and changing its lignocellulolytic activity to match the plant substrate available. The selection of plant polymers degraded by *L. gongylophorus* may also depend on the nutritional status of the host colony, as more recalcitrant polymers present a vast food supply but require the input of additional resources for effective degradation. 

A potential explanation to the limited lignocellulolytic capacity of *L. gongylophorus *pure cultures is that the host ants enhance the biodegradative capacity of this organism *in situ*. Attines are known to concentrate lignocellulolytic enzymes in fecal droplets that they deposit on freshly integrated plant biomass in fungus gardens, presumably to assist in the first stages of biomass degradation, or, as mentioned above, to inhibit potential garden pests [77,107,108,109]. Recent work has confirmed these enzymes originate from the fungal gongylidia consumed by the ants, and that a variety of pectinases, carboxymethylcellulases, amylases, and even laccases are present in this cocktail [63,71]. The relative importance of this pre-treatment step to overall biomass degradation in fungus gardens is not known, nor is the full extent of the enzymes that may be present in fecal droplets. 

It is also possible that microbes other than the fungal cultivar are essential for plant biomass degradation, either by deconstructing plant polymers directly or by enhancing the lignocellulolytic activity of the fungal cultivar. There is precedent for bacterial-fungal interactions promoting fungal lignocellulose degradation in other systems, but the mechanisms for these phenomena remain unclear [110]. Lignocellulolytic bacteria have also been cultured directly from fungus gardens [58], indicating that these microbes could participate directly in biomass processing. Recent metagenomic analyses have confirmed that bacteria in fungus gardens possess a variety of glycoside hydrolase genes that could potentially deconstruct plant polysaccharides directly [44,45], and proteomic work has confirmed that at least some of these genes are expressed in fungus gardens [45]. A number of studies have investigated microfungi and yeasts in fungus gardens (Table 1), but the lignocellulolytic capacities of these organisms have yet to be investigated. 

Regardless if specific microbe-microbe or ant-microbe interactions are significantly influencing biomass degradation, it is clear that the physiology of fungus garden microbes in pure culture do not fully reflect their *in situ* physiology. The diverse lignocellulolytic activities repeatedly measured from both *L. gongylophorus *and directly from fungus gardens indicate that a wide variety of plant polymers, including cellulose, are likely degraded in this ecosystem [44,57,61,62,68,69,70,72,74]. However, the amount of degradation may vary greatly depending on the foliar biomass harvested by the ants and other community-level processes that have yet to be elucidated [62,70,72]. Confirming the extent to which the fungal cultivar, resident bacteria, and other microbes in fungus gardens contribute to lignocellulose degradation, and more thoroughly characterizing the *in situ* physiology of this ecosystem, remains an important future direction in this field. 

## 6. Reciprocal Adaptation of the Ant Genome

The application of sequenced-based approaches to investigate the fungus-growing ant system has advanced to include the ants themselves. The recently sequenced genomes of the leaf-cutter ants *Atta cephalotes* and *Acromyrmex echinatior* have provided a wealth of information for studying the symbiosis between these ants and their fungus gardens [17,18]. The obligate dependence of these ants on their cultivated fungi has been thought to lead to reductions at the genomic level, as has previously been observed in other model nutritional symbiosis such as between the pea aphid and its nutrient-producing bacterial endosymbionts [111]. In the pea aphid system, the host genome was found to lack genes for the biosynthesis of specific amino acids known to be produced by its endosymbiont *Buchnera* [111,112]. Similarly, both genomes of the leaf-cutter ants were found to lack genes required for arginine biosynthesis, in contrast to other ant genomes that contain the entire pathway [17,18]. One hypothesis is that the fungus may provide arginine to the ants, thereby limiting the need for this particular pathway. Previous work documenting the compounds in an *Atta colombica* cultivar has shown the presence of free arginine, providing some support for this hypothesis [101]. The *A. cephalotes* genome was also found to be missing a hexamerin gene thought to be involved in amino acid sequestration during larval development [18], potentially indicating that these ants have a reduced need to store amino acids since larvae may get these nutrients from the fungal cultivar.

Serine proteases, which are potentially important in the degradation of proteins in the diet, were also found to be reduced in the *A. cephalotes* genome compared to other insects [18]. As with the loss of arginine biosynthesis and hexamerins, this may reflect a decreased capacity of leaf-cutter ants to acquire nutrients from their environment coincident with their dependence on *L. gongylophorus* for food. Another possible hypothesis is that this reduction in proteases is related to the ants’ ability to concentrate fungal enzymes in their fecal droplets. Because extensive degradation of proteins in the diet would preclude the concentration and application of fungal enzymes to the fungus gardens, it is possible that the lack of these genes is a result of millions of years of this peculiar behavior. 

Overall these data are consistent with the hypothesis that fungus-growing ants have lost some capacity to acquire nutrients over the course of their 50 million year co-evolutionary history with their obligate fungal mutualist. It is interesting to note that co-evolved symbioses, characterized by the evolutionary innovation provided by both partners, appear to be accompanied by physiological restrictions in the hosts. Perhaps most striking is the possibility that a behavioral innovation of the ants—the concentration of lignocellulolytic and potentially antimicrobial fungal enzymes in fecal droplets—may have also resulted in profound genomic changes. These results, together with what is known for other symbioses such as the pea aphid-*Buchnera* system, suggest a common theme in nutritional symbioses: prolonged reliance by the host on a single symbiont for nutrition resulting in extensive and elaborate evolutionary transitions towards obligate association. 

## 7. Conclusions and Future Outlook

Attine ants have long been a model system for the study of symbiosis, co-evolution, and evolutionary innovation. The intriguing symbiosis between these ants and their fungus-bacteria consortia is remarkable both because of its stability (~50 million years) and the drastic effects it has had on both partners (production of hyphal swellings and shifts in enzymatic profiles by the fungal cultivar, and genomic and behavioral changes in the host ants). Here we have reviewed how advances in genome sequencing and culture-independent investigations of microbial communities, together with more traditional approaches, have transformed our understanding of co-evolution and evolutionary innovation in the fungus-growing ants. Future work answering key questions of plant biomass degradation and nutrient conversion in fungus gardens, reciprocal ant-fungus co-evolution, and fungus garden microbial diversity will allow for a more fundamental understanding of this remarkable system. For example, advances in proteomics and *in situ* analyses of microbial communities will shed light on the degradation of cellulose in fungus gardens, and the role different microbial groups play in biomass processing. Moreover, by leveraging the large amount of sequence information now available for the organisms in this symbiosis, it will soon be possible to understand the degree of metabolic integration between the ants and their fungus, and the extent to which the behavior of the host has been altered by genetic and physiological constraints of the symbiosis. Given the prevalence of symbiotic microbial communities in nature, gaining a fundamental understanding of the ant-fungus garden system will likely have far-reaching implications for understanding broader trends in the ecology and evolution of metazoans. 
